# Mapping awareness-raising and capacity-building materials on developmental disabilities for non-specialists: a review of the academic and grey literature

**DOI:** 10.1186/s13033-024-00627-9

**Published:** 2024-02-24

**Authors:** Elisa Genovesi, Yuan Ishtar Yao, Emily Mitchell, Michal Arad, Victoria Diamant, Areej Panju, Charlotte Hanlon, Bethlehem Tekola, Rosa A. Hoekstra

**Affiliations:** 1 Department of Psychology, Institute of Psychiatry, Psychology and Neuroscience (IoPPN), King’s College London, Addison House, Guy’s Campus, London, SE11UL UK; 2 Centre for Global Mental Health, Department of Health Services and Population Research and WHO Collaborating Centre for Mental Health Research and Training, Institute of Psychiatry, Psychology and Neuroscience (IoPPN), King’s College London, 16 De Crespigny Park, London, SE58AB UK; 3 Department of Psychiatry, WHO Collaborating Centre for Mental Health Research and Capacity-Building, School of Medicine, College of Health Sciences, Addis Ababa University, Addis Ababa, Ethiopia; 4 Centre for Innovative Drug Development and Therapeutic Trials for Africa, College of Health Sciences, Addis Ababa University, Addis Ababa, Ethiopia

**Keywords:** Grey literature, Capacity building, Developmental disabilities, Autism, Low- and Middle-Income Countries

## Abstract

Most children with developmental disabilities (DD), such as intellectual disabilities and autism, live in low- and middle-income countries (LMICs), where services are usually limited. Various governmental, non-governmental and research organisations in LMICs have developed awareness-raising campaigns and training and education resources on DD in childhood relevant to LMICs. This study aimed to comprehensively search and review freely available materials in the academic and grey literature, aimed at awareness raising, training and education on DD among non-specialist professionals and community members in LMICs. We consulted 183 experts, conducted key-word searches in five academic databases, four grey-literature databases and seventeen customised Google search engines. Following initial screening, we manually searched relevant systematic reviews and lists of resources and conducted forwards and backwards citation checks of included articles. We identified 7327 articles and resources after deduplication. We then used a rigorous multi-step screening process to select 78 training resources on DD relevant to LMICs, of which 43 aimed at informing and/or raising awareness DD, 16 highlighted specific strategies for staff in health settings and 19 in education settings. Our mapping analysis revealed that a wealth of materials is available for both global and local use, including comics, children’s books, flyers, posters, fact sheets, blogs, videos, websites pages, social media channels, handbooks and self-education guides, and training programmes or sessions. Twelve resources were developed for cross-continental or global use in LMICs, 19 were developed for and/or used in Africa, 23 in Asia, 24 in Latin America. Most resources were developed within the context where they were intended to be used. Identified gaps included a limited range of resources on intellectual disabilities, manuals for actively delivering training to staff in education settings and resources targeted at eastern European LMICs: future intervention development and adaptation efforts should address such gaps, to ensure capacity building materials exist for a sufficient variety of DD, settings and geographical areas. Beyond identifying these gaps, the value of the review lies in the compilation of summary tables of information on all freely available resources found, to support their selection and use in wider contexts. Information on the resource content, country of original development and copyright is provided to facilitate resource sharing and uptake.

**Supplementary Information:**

The online version contains supplementary material available at 10.1186/s13033-024-00627-9.

## Background

Developmental disabilities (DD), such as intellectual disabilities and autism, are conditions associated to lifelong difficulties in communication and/or cognition [37]. Most children with DD^1^ live in low- and middle-income countries (LMICs; [21], where their needs are often unmet due to a shortage of services. A major barrier to service delivery is the scarce availability of trained professionals and suitable programmes for raising awareness on DD and reducing community stigma [2, 36]. A recent review of reviews on autism services suggested that a lack of awareness in the community and health systems [1] and of culturally appropriate and valid identification tools [30] causes a “detection gap” in LMICs, whereby signs of autism are undetected or recognised at a later age than in high income countries [6]. Similarly, the scarcity of specialist services is responsible for a “care gap” [25], aggravated by limited inclusion in the community and support by community members. This care gap is often due to a lack of appropriate training and stigma on the part of the service provider resulting in poor understanding of the child’s needs and of possible intervention strategies [1, 2].

In 2013, the World Health Organization called for an increase in international activities for public awareness raising and capacity building of professionals to better address the needs of children with DD in LMICs [36]. Capacity building was to be especially directed at non-specialists, as is the case in task-sharing interventions, largely used in global health to increase service provision in the face of scarcity of specialist health practitioners [24]. Divan et al. [6] further highlighted the need for training health and social care workers and educators to integrate support services for autism in existing services. Integration of care into various platforms is also recommended to address children’s mental health needs more generally (Kieling et al. 2011). Raising awareness in the broader community is equally important to reduce barriers to inclusion and services for children with DD [1, 2, 5, 28].

Various governmental, non-governmental and research organisations in several LMICs have developed awareness raising campaigns related to DD, as well as programmes and materials for the education (informational and mostly theoretical learning) and training (practical learning of relevant skills), henceforth referred as training resources. For example, in the Health Education and Training HEAT + project in Ethiopia [34], audio-visual and written training materials focused on DD were developed and added to a previously used mental health training programme for non-specialist health workers. The mental health manual for health workers “Where there is no psychiatrist” [24] includes a section on DD, which describes common signs to support identification, as well as providing guidance on referring children to specialised services and supporting caregivers in their child’s care. Similarly, the manual “Educating and caring for children with profound intellectual disability” developed in South Africa as part of the Teacher Empowerment for Disability Inclusion project [32] can be used to educate schoolteachers on DD and strategies to effectively include children with DD in their teaching practices. The video “Recognising autism” by Sangath in India [27] is an example of a wide-reaching awareness raising campaign aimed at promoting early detection of autism in the community.

While the development and testing of some training resources have been documented thoroughly (e.g., [32, 34]), other materials developed and used by grassroot organisations are less well-known and only available in non-peer-reviewed resources (e.g., [12, 35]). These resources are typically developed and used in only one country or region. An effort to document multiple materials appropriate for LMICs from several sources is critical to promote mutual learning across organisations and provide a comprehensive understanding of previously used resources. Such understanding could allow for existing resources to be adapted and implemented in different contexts from where they were originally developed, hence widening the reach of awareness and capacity building interventions and preventing unnecessary duplication of efforts and expense.

The aim of this study was to comprehensively search and review materials in the academic and grey literature that are or can be made freely available, aimed at one or both these functions: i.e. raising awareness of DD among non-specialist professionals and community members in LMICs and/or building their capacity to increase acceptance of children with DD in the community and promote their inclusion in the society and access to services (e.g. health and education services). Grey literature encompasses any document beyond academic articles published in peer-reviewed journals and commercial publications, such as research reports which are not peer-reviewed, as may often be the case for authors and institutions in LMICs with limited capacity to publish through commercial publication routes, as well as policy documents and informational reports, manuals, materials produced for use by non-governmental organisations, web pages, etc. [7]. Collating these documents for review involves, as well as searching specific databases of grey literature, the exploration of other sources, such as relevant websites, and solicitation of suggestions from experts in the field [10]. While some of these search methods may be less systematic than academic database searches, including grey literature allows for comprehensiveness in the review, and methodological choices can be made systematically and be rigorous [10].

We will describe overall patterns and gaps in available materials, and document the main features of the resources identified, in order to provide clear information to organisations and teams who wish to select the most appropriate resource to adapt to their project and context.

## Methods

### Search strategy

The review followed the Preferred Reporting Items for Systematic Reviews and Meta-Analyses (PRISMA) guidelines [23] as well as specific guidance for searching grey literature [10].

The search, conducted between June and October 2021, combined four methods—expert consultations, academic database search, grey literature database search, customised Google search engines—and was supplemented by manual search of relevant systematic reviews and lists of resources and forwards and backwards citation checks of included articles.

The full search strategy, including all terms, is available in Additional file 1.

### Expert consultations

**Fig. 1:**
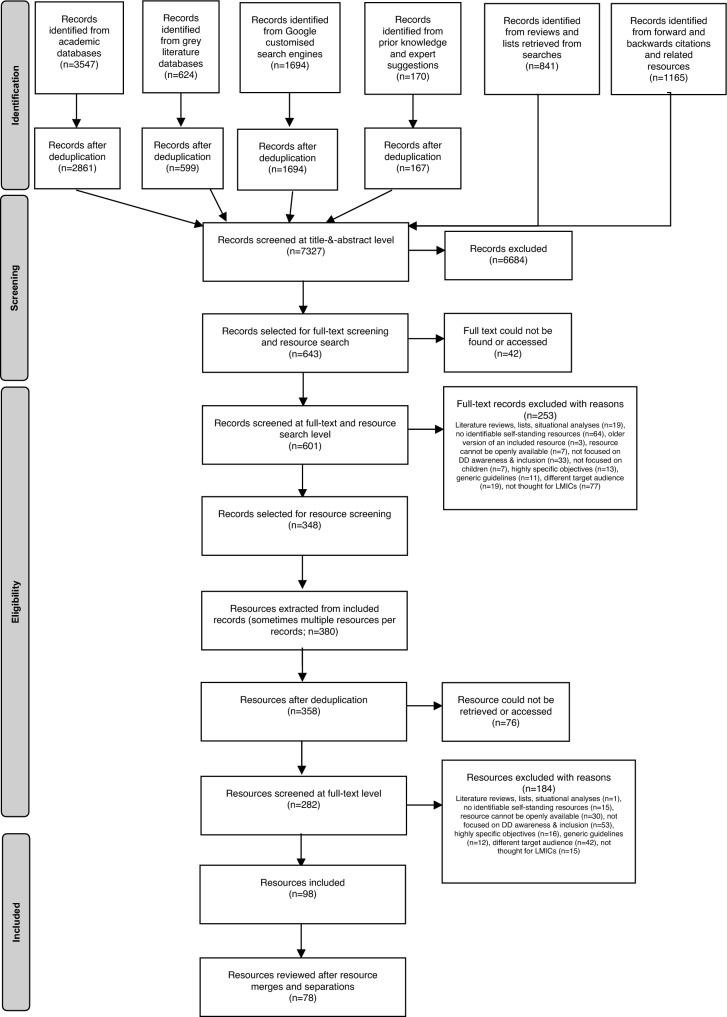
PRISMA flow diagram (Moher et al. 2009) of the study selection process

A list of disability and inclusive education experts was compiled, aiming for comprehensive coverage of experts working in LMICs in different continents. Experts were contacted via email with a brief description of the aims of the review and asked to provide suggestions on relevant resources. A follow up email was sent 2–4 weeks after first contact to those who had not replied. We compiled a spreadsheet of all suggestions received, including recommendations of additional experts, whom we then emailed. Of 183 experts contacted in total, 91 replied. These experts made suggestions relating to 162 resources. Eight further resources were suggested by experts within the research team (total n = 170, see Fig. 1).

### Academic database search

Four academic databases (PsycInfo, MEDLINE, Embase, Global Health) were searched on the Ovid platform and one (ERIC) on the Ebsco platform.

The search utilised search truncated terms for greater comprehensiveness and combined terms related to:

- DD and special needs (the latter more general term was included as it is often used to describe disabilities in education settings, excluding technical terms of specific diagnoses)
- Training resources
- Non-specialist audiences
- LMICs

In Ovid, where multiple databases had been searched simultaneously, results were deduplicated automatically. All records were then exported in Excel from both platforms.

### Grey literature database search

Four grey literature databases were searched separately online. Searches adopted slightly different methodologies depending on the structure and content of the database: for example some allowed combining search terms while in others only one term at a time could be searched, some automatically searched plural and singular forms together and in others both forms had to be entered separately, etc. Nonetheless, search strategies aimed for the greatest possible consistency, minimising any differences in search comprehensiveness. While truncation could not be easily used in some of these databases, several relevant variations of search terms were used when the database did not automatically include word variations. Additional file 1 details each search strategy used.

The World Health Organisation IRIS, the UNESCO Library, and Eldis were searched only with DD/ “special needs” terms. APA PsycExtra was searched by combining DD/ “special needs” terms with terms for LMICs, as the database, differently from the previous ones, does not have a clear global focus. All records were exported in Excel.

### Customised google searches

The research team drew from experience, expert suggestions, and web searches in order to compile a list of websites of relevant NGOs (e.g., disability NGOs, education NGOs) operating in LMICs, either at country or region level or internationally. Seventeen customised Google engines were then created as follows:

- One for World Health Organisation website (who.int)
- One for UNICEF website (unicef.org)
- One for Autism Speaks website (autismspeaks.org)
- Two for 16 Africa-based NGO websites
- Two for 12 Latin America-based and 1 Eastern Europe-based NGO websites combined
- Three for 16 Asia-based NGO websites
- One for 8 international education NGO websites
- An additional 60 websites were randomly assigned to 6 more engines

The unequal distribution of websites was carefully planned to avoid getting results only from a small number of highly influential websites. For each engine, several relevant variations of DD/special needs terms combined with OR were entered in the search box and the results, automatically limited by Google to 100 records per engine, were exported manually in Excel.

### Screening process

Records were screened in three stages: title (and abstract when available) screening, full-text screening of articles and identification of potentially relevant resources, and finally selection of relevant resources among the ones identified. Some individual records described multiple training resources, leading to 380 resources being extracted from the 348 originally included records (see Fig. 1). At all stages, 20% of records were double-screened to define inclusion/exclusion criteria and check for consistency and accuracy in the process. Disagreements were resolved through discussions between reviewers and with the team.

The finalised inclusion criteria used throughout screening of all resources were as follows:

1. Self-standing resources that can be used for direct (self-)education of non-specialists and aimed at increasing their awareness and inclusion skills, rather than academic knowledge. These include flyers, educational book chapters, manuals, guidelines for non-specialists, online training programmes, websites, etc. These were retrieved separately or identified through studies, initiatives, projects, or project protocols focused on their development, testing, use, implementation or scale-up.
2. Resources that can be made available openly and freely, while not necessarily being readily and publicly accessible online at the time of the review.
3. Resources with a non-specialist target audience; for the purpose of this review, specialists are any individuals with a Master’s or specialisation degree, or above qualification, in a discipline relevant to developmental disabilities, including special needs education, paediatrics, mental health and rehabilitation specialisms. The target audience for the current review was anyone without these relevant qualifications, including but not limited to non-specialist teachers, non-specialist health workers and medical doctors and the community at large.
4. Resources focused on (or including as one of the main modules) a description of characteristics, causes, prognosis etc. of DD or on strategies for including children DD in the community or engaging and educating children with DD in schools. For the purposes of this review, DD include autism, intellectual disabilities, language and social communication disabilities (Stein et al., 2020). We included resources on specific conditions, such as cerebral palsy, consistently associated with intellectual disability and communication difficulties, when the resource was focused on the cognitive and communication challenges rather than associated physical challenges. Resources relevant to developmental delays that are not yet formally clinically diagnosed were also included.
5. Resources focused on children, infants and toddlers, as defined by the study or resource itself, or relevant to children, infants and toddlers, when no age group was specified.
6. Resources developed and/or used in a LMIC, as defined by the World Bank Group (2021) or resources that appear to have been developed in high-income countries for global use with a particular focus on LMICs in mind.

The exclusion criteria used were the following:

1. Literature reviews, situational analyses and general reports or guidelines giving broad directions/indications, rather than informing on diagnostic characteristics, causes, prognosis etc. of DD or on strategies for engaging and educating children with DD in schools/ or supporting children in the community.
2. Non-manualised training programs or sessions, with any available resources non-specific and not self-standing.
3. Protocols on intervention development for which the training programme does not yet exist.
4. Resources aimed at family members to improve their relationships with and/or care for children with DD in their family, or at children with DD themselves.
5. Resources focused only on training teachers on a very specific strategy not widely applicable to inclusive teaching (e.g., drawing, a specific social game, etc.) or training health workers on a very specific clinical skill (e.g. using an identification checklist), or is relative to a specific period or event (e.g., COVID-19 pandemic), and/or addresses a very specific skill of children with developmental disabilities, e.g., a school intervention aimed at improving autistic children’s spelling ability.
6. Resources focused only on one or more Specific Learning Disabilities (i.e. dyslexia, dyscalculia, dyspraxia, etc.) that are not one of the key DD previously specified.
7. Subsequently, resources that were no longer available online upon manuscript submission in April 2023 were removed prior to submission.

### Mapping

Before final extraction of the information, resources were sometimes grouped together (e.g., instead of multiple videos from one social media channel we included the whole channel if it was relevant overall) or split (e.g., in a handbook with several irrelevant chapters and a few relevant distinct chapters we treated each relevant chapter as an individual resource). The following information was extracted for each resource, from the resources themselves and from any articles discussing them in the originally screened records: author, date, country/ies of origin and/or use, format, mode of communication, target audience, content (type of DD, topic), objectives, inclusivity and consideration of culture and context in development, mode and time of delivery and additional implementation features, how the resource was tested, surprising or unusual content and features. The availability of such information varied.

As well as providing a brief summary of each resource, the information extracted was used to give an overview of the available resources in terms of evidence, features, and content, and identify any gaps for future work.

## Results

After screening 7327 records and 380 resources, a total of 78 resources were selected, of which 10 were accompanied by at least one research report or media article retrieved in the search. Information on the resources identified in each phase is reported in the PRISMA flow diagram [23] in Fig. 1. The tables provide a summary of extracted information for each resource, distinguishing among those providing general information on DDs and inclusion and/or aimed at raising awareness (Table 1; 43 resources), and those highlighting specific strategies for staff in health settings (Table 2; 16 resources) and education settings (Table 3; 19 resources). Additional file 2 provides more detailed information on such resources.

**Table 1:** Summary of resources for general awareness raising

Title	Organisations/Authors	Date produced	Langauge(s)	Type/format of resource	Where was it developed	For use where?	Audience	DD targeted	Age group targeted
Recognising autism	SangathGoa	2018	Hindi with English subtitles	Video	India	India	General public	Autism	Childhood
Autism by Zemi Yenus—Babiye and autism	Zemi Yenus (speaker); Tsehai Loves Learning (producer)	2021	Amharic	Video	Ethiopia	Ethiopia	General public	Autism	Below 5 years old
Entendendo Autismo (understanding autism)	Entendendo Autismo	Created 2010, last updated 2022	Portuguese	Facebook page of virtual flyers	Brazil	Brazil	The community, children peers, teachers, (parents)	Autism	Childhood, (adolescence, adulthood)
We'll make it	Author: Pierre Gento, Artist: Vincent Ringler Producer: Sanofi	2013	English, French Amharic	Comic book	Kenya	Africa	School pupils and students, teachers, nurses, community/religious leaders, general public	Epilepsy	Unspecified school age, relevant to childhood
demystifying autism	Saima Wazed Hossain, Shuchona Foundation	Unavailable	English	Blog	Bangladesh	Bangladesh	General public	Autism	Unspecified, relevant to childhood
Developmental disabilities (in “resources”)	Institute of Paediatric Neurodisorder & Autism (IPNA)	Unavailable	English	Fact sheets	Bangladesh	Bangladesh	General public (with some pages that seem directly specifically at parents)	Autism, ID, cerebral palsy, down syndrome, William syndrome	Childhood
Developmental disability	Ubuntu-Hub (Juntos team)	Unavailable; The project started in 2017	English, Portuguese, Spanish	Video	United Kingdom	Colombia, Brazil; Ubuntu Hub resources are now being used in several LMICs	General public	All DD	Unspecified
Autistologos.com	Autistologos	Unavailable	Portuguese	Website with several relevant blogs	Brazil	Brazil	General public	Autism	Childhood
Biblioteca virtual brincar	Fundación Brincar	Unavailable	Spanish	Online library with several relevant blogs	Argentina	Argentina	General public	Autism	Childhood (and adolescence and adulthood)
Mental retardation: from knowledge to action	Dr. Satish Girimaji, Dr Sultana S. Zaman, Mrs. P.M. Wijetunga, Dr. Udom Pejarasangharn	2001	English	Handbook	India	South-East Asia	Policy-makers, caregivers, and other carers (presumably professionals, community members)	ID	Unspecified, substantial focus on childhood
Autismo: Lidando com comportamentos socialmente inadequados (Autism: dealing with socially inappropriate behaviour)	Juliana Fialho	2012	Portuguese	Blog	Brazil	Brazil	Education and health professionals (and caregivers)	Autism	Childhood
Childhood Neurodevelopmental Disorders (CNDD)	Muideen O. Bakare, Mashudat A. Bello-Mojeed, Kerim M. Munir, Oluwayemi C. Ogun, Julian Eaton and MosunmolaF. Tunde-Ayinmode	2016	English	Flyer	Nigeria	Nigeria	Teachers and parents	Down Syndrome, cerebral palsy, autism, language disabilities, Fragile-X Syndrome	Childhood
What is CP? Social story (in Co-designing health and education materials)	Centre for Augmentative and Alternative Communication, University of Pretoria	2020	Afrikaans, English, isiZulu, seSotho sa-lebowa, seTswana	Video	South Africa	South Africa and more broadly in Africa	Healthworkers	Cerebral palsy, focus on communication disability	Childhood
Detección Temprana (Early detection)	PANAACEA	Unavailable	Spanish	Blog	Argentina	Argentina	General public	Autism	0–48 months
PANAACEA	PANAACEA (Asociación Civil Programa Argentino para Niños, Adolescentes y Adultos con Condiciones del Espectro Autista)	Created 2011, last updated 2022	Spanish	YouTube channel	Argentina	Argentina	For videos explored: teachers, community members (and parents)	Autism	Childhood, (adolescence, adulthood)
ALUZAZUL	ALUZAZUL	Created 2016, last updated 2021	Portuguese	YouTube channel	Brazil	Brazil	Professionals (and caregivers)	Autism	Infancy, childhood, (adolescence and adulthood)
Istituto Farol—Autismo (Instituto Farol- autism)	Thiago Lopes (Instituto Farol)	Created 2018, last updated 2022	Portuguese	YouTube channel	Brazil	Brazil	Professionals (and caregivers)	Autism	Infancy, childhood, (adolescence and adulthood)
André e o autismo (André and autism)	MSP—Mauricio de Sousa Produções	2019	Portuguese	6-episode series of videos	Brazil	Brazil	The community, teachers (and parents)	Autism	Childhood
Mayra Gaiato | Autismo e Desenvolvimento Infantil (autism and childhood development)	Mayra Gaiato	Created 2016, last updated 2022	Portuguese	YouTube channel	Brazil	Brazil	For videos explored: teachers, community members (and parents)	Autism, ID	Infancy, childhood, (adolescence and adulthood)
Luna ABA	Luna ABA	Created 2010, last updated 2022	Portuguese	YouTube channel	Brazil	Brazil	For videos explored: teachers (and parents)	Autism, ID, other DD	Childhood, (adolescence and adulthood)
Willian Chimura	Willian Chimura	Created 2019, last updated 2022	Portuguese	YouTube channel	Brazil	Brazil	For videos explored: the community	Autism	Childhood, (adolescence and adulthood)
Autismo: compreensão e práticas baseadas em evidências (Autism: understanding and evidence-based practices)	Paulo Liberalesso e Lucelmo Lacerda (Movimento Capricha na Inclusão)	2020	Portuguese	Handbook	Brazil	Brazil	Public administrators, politicians and teachers	Autism	Childhood, (adolescence and adulthood)
Iluminemos Por el Autismo	Iluminemos Por el Autismo	Created 2015, last updated 2022	Spanish	YouTube video	Mexico	Mexico	General public	Autism	Unspecified
Ummeed child development center youtube channel	Ummeed	2016	Hindu, at times with English subtitles	YouTube channel	India	India	Teachers, community members (and mostly caregivers)	General DD	Childhood
Autism spectrum disorders	World Health Organization	Last updated 2021	English, French, Spanish, Arabic, Chinese, Russian	Fact Sheet	Switzerland	Global	General public	Autism	Unspecified
Información (information)	RedEA	2014–2022	Spanish	Fact Sheets and blogs	Argentina	Argentina	General public, teachers, (caregivers)	Autism	Childhood
About autism	Action for Autism	Unavailable	English	Fact Sheet	India	India	General public	Autism	Childhood, from 18 months
Watch for signs of autism: detect early	Action for Autism	Unavailable	Hindi, English	Poster	India	India	Unavailable information	Autism	Childhood
Practical guide to autism spectrum disorder (and supplement: autism spectrum disorder, high functioning)	Autism South Africa	Unavailable	English, Sepedi, Zulu, Tswana, Sesotho, Xhosa, Afrikaans	Handbook (with supplementary handbook)	South Africa	South Africa	Unavailable information	Autism	Unspecified
Early years and autism spectrum disorders	Christine Deudney and Lynda Tucker	2010	English	Handbook	UK and South Africa	South Africa	Unavailable information	Autism	Early childhood
Why does Chris do that? Some suggestions regarding the cause and management of the unusual behaviour of children and adults with autism and Asperger syndrome	Tony Attwood	2010	English	Handbook	UK and South Africa	South Africa	Professionals, (parents, and carers)	Autism	Childhood
The sensory world of the Autistic spectrum: a greater understanding	Kate Wilkes	Unavailable	English	Handbook	UK and South Africa	South Africa (through Autism South Africa)	General public	Autism	Unspecified
The super useful guide to managing meltdowns	Bec Oakley	2013	English	Handbook (guide)	South Africa	South Africa (through Autism South Africa)	Teachers, caregivers, general public	Autism, ID, communication disabilities, other DD	Childhood, adloescence, (adulthood)
Kit de ferramentas para comportamentos desafiadores e agressivos (Challenging and aggressive behaviours toolkit)	Autism Speaks (translated by Autismo e Realidade)	2012	Portuguese	Handbook (toolkit)	USA	Brazil	Teachers, helthworkers, (parents and caregivers)	Autism	Challenging and aggressive behaviour and strategies to manage it
Manual para as escolas (Manual for schools) school community tool kit	Autism Speaks (translated by Autismo e Realidade)	2011	Portuguese	Handbook (manual)	USA	Brazil	School community (teachers, administrators, aides, office staff, bus drivers, nurses, custodians, peers and parents)	Autism	Childhood, school age
Guia para Leigos sobre o Transtorno do Espectro Autista (TEA) (guide for laypeople on Autism spectrum disorder)	Autismo e Realidade	2021	Portuguese	Handbook (guide)	Brazil	Brazil	Lay public interested in autism, (and caregivers)	Autism	Childhood (and adolescence and adulthood)
Cartilha Autismo: uma realidade (autism booklet: a reality)	Autismo e Realidade	2013	Portuguese	Comic book	Brazil	Brazil	Teachers, health professionals (and parents)	Autism	Childhood, school age
ICO e o Mundo Que Queremos Construir (Ico and the World we want to build)	Carina Alves & Elyse Matos	2020	Portuguese	Children's book	Brazil	Brazil	"Lay public, or ""Everyone who wants to think differently to change the world”	Autism	Childhood (10yo)
Diferenças entre TEA (Transtorno do Espectro Autista) e TDL (Transtorno do Desenvolvimento da Linguagem) (Differences between autism and communication development disability)	Mundo TDL	2019	Portuguese	Poster/illustrative table	Brazil	Brazil	Lay public and Equipemundo's followers	Autism, language disability	Unspecified
The RehApp: cerebral palsy	Enablement	Unavailable	English, French, Nepali, Portuguese, Khmer	Chapter in online app	The Netherlands	Low- and middle-income countries (especially in Africa, South America and south East Asia)	Fieldworkers	Cerebral palsy	Unspecified overall, but with specific references to babies and children
The RehApp: intellectual disability	Enablement	Unavailable	English, French, Nepali, Portuguese, Khmer	Chapter in online app	The Netherlands, Indonesia	Low- and middle-income countries (especially in Africa, South America and south East Asia)	Fieldworkers	ID	Unspecified overall, but with specific references to babies and children
The RehApp: autism spectrum disorder	Enablement	Unavailable	English, French, Nepali, Portuguese, Khmer	Chapter in online app	The Netherlands	Low- and middle-income countries (especially in Africa, South America and south East Asia)	Fieldworkers	Autism	From birth to adulthood
The RehApp: down syndrome	Enablement	Unavailable	English, French, Nepali, Portuguese, Khmer	Chapter in online app	The Netherlands	Low- and middle-income countries (especially in Africa, South America and south East Asia)	Fieldworkers	Down Syndrome	Unspecified overall, but with specific references to babies and children

**Table 2:** Summary of resources for health settings

Title	Organisations/Authors	Date produced	Langauge(s)	Type/format of resource	Where was it developed	For use where?	Audience	DD targeted	Age group targeted
When Children Have Problems (Study Session 17 in the Non-Communicable Diseases, Emergency Care and Mental Health Module of the Health Education and Training HEAT programme)	The Open University (UK), Addis Ababa University (Ethiopia), Ethiopian Federal Ministry of Health	2013	English, Ahmaric	Training session	UK, Ethiopia	Ethiopia; can be used across sub-Saharan Africa	Health extension workers	ID, mentions of other DD	Childhood (and adolescents)
Child developmental and mental health problems (page 21 and chapter 6 in Mental Health: a Guide for Community Health Workers of the Health Education and Training in the HEAT + programme)	The Open University (UK), Addis Ababa University (Ethiopia)	2017	English, Ahmaric	Handbook (pocket guide)	UK, Ethiopia	Ethiopia, adaptable to other contexts	Health extension workers	ID, autism, other DD	Childhood
Training videos on childhood developmental problems (in the Non-Communicable Diseases, Emergency Care and Mental Health Module of the Health Education and Training HEAT + programme)	The Open University (UK), Addis Ababa University (Ethiopia)	2017	Amharic, Amharic with English subtitles	Videos	UK, Ethiopia	Ethiopia, adaptable to other contexts	Health extension workers	ID, autism	Childhood
Problems in childhood and adolescence (chapter 11 in where there is no psychiatrist)	Vikram Patel, Charlotte Hanlon	2018	English	Book/guide	UK, USA, Ethiopia	Globally, especially LMICs and low-resourced settings	General health workers	ID, cummunication and learning disabilities, autism	Childhood (and adolescence)
Therapeutic Intervention for autism (TIA) clinic (Tutorial 5.4, pp 669–729, in Training manual for the establishment of child development and disability services in Bangladesh)	Bangladesh Protibondhi Foundation	2021	English	Training tutorial in multi-professional training programme (manual)	Bangladesh	Bangladesh	Generic health workers (and developmental therapists)	Autism	0–18 years (and adulthood)
Speach, Language and Communication (SLC) clinic (Tutorial 5.5, pp 730–766, in Training manual for the establishment of child development and disability services in Bangladesh)	Bangladesh Protibondhi Foundation	2021	English	Training tutorial in multi-professional training programme (manual)	Bangladesh	Bangladesh	Generic health workers (and developmental therapists)	Speech, communication disabilities	0–18 years (and adulthood)
Well Baby and Neonatal Clinic (WBC) (Tutorial 5.6, pp 767–789, in Training manual for the establishment of child development and disability services in Bangladesh)	Bangladesh Protibondhi Foundation	2021	English	Training tutorial in multi-professional training programme (manual)	Bangladesh	Bangladesh	Generic health workers (and developmental therapists)	General DD	0–5 years
Child&Adolescent Mental & Behavioural Disorders (CMH, pp. 69–92 in mhGAP Intervention Guide 2.0)	World Health Organization Department of Mental Health and Substance Abuse	2016	English, Arabic, French, Italian, Marathi, Russian, Spanish, Thai, Ukrainian	Handbook (decision-making tool)	Switzerland	LMICs, extended to all World Health Organization regions	Non-specialist health workers	Behaviour problems, general DD	0–12 years (and adolescence and adulthood)
Child and adolescent mental and behavioural disorders (module in the mhGAP Training of Health-care providers (TOHP))	World Health Organization	2017	English	Training programme module	Switzerland	LMICs	Non-specialist health-care providers	General DD, autism, ID	Childhood (and adolescence)
Signs of Mental retardation	Geeta Chopra	pre-1999	English, Hindi	Poster	India	India	Community child care and health workers	ID	3 months—5 years
Managing a child with intellectual challenge: general principles	Geeta Chopra	pre-1999	English, Hindi	Poster	India	India	Community child care and health workers	ID	Childhood
Caring for children with development disabilities	Multi-Agency International Training and Support	2017	English (available in Nepalese upon request)	Handbook (guide, manual)	United Kingdom	India and other LMICs in Asia; it can be used in low-resource settings globally (according to the resource)	Non-specialist health workers who have received previous informal training on DD (e.g. MAITS Working with Children with Developmental Disabilities and their caregivers—for non-specialists in low resource settings)	Cerebral palsy, ID, autism, epilepsy	Birth to adolescence
Working with children with developmental disabilities and their caregivers—for non-specialists in low resource settings	Multi-Agency International Training and Support	2018	English	Training programme (manual and presentation)	United Kingdom	Low-resource settings	Non-specialist health workers who have experience of working with children, and preferably some experience of working with children with disabilities, but have little or no knowledge of developmental disability	Cerebral palsy, spina bifida, ID, autism, epilepsy	Childhood, mostly 0–8 years
Intellectual disability. A manual for CBR workers	Jayanthi Narayan; published by World Health Organization Regional Office for South-East Asia	2007	English, Telugu	Handbook / training manual	India	India, with possibility to use in Member Countries of the World Health Organization South-East Asia Region	Community based rehabilitation workers	ID	Childhood, (adolescence and young adulthood)
Mental Retardation (Chapter 32 in disabled village children. A guide for community health workers, rehabilitation workers, and families.)	David Werner	1987	English	Chapter in an interactive book (guide)	USA	Global, especially in rural areas	Community health workers, rehabilitation workers, and families	ID	Childhood
Patients with an autism spectrum disorder—information for health professionals	Christine Deudney	2010	English	Handbook	UK and South Africa	South Africa	All health professionals and hospital who may come into contact with an adult or child with autism for reasons other than their autism	Autism	Childhood (and adulthood)

**Table 3:** Summary of resources for education settings

Title	Organisations/Authors	Date produced	Langauge(s)	Type/format of resource	Where was it developed	For use where?	Audience	DD targeted	Age group targeted
The story of Khamdy	Hui Min Low and associates	2019	English, Laotian	Online teacher training module	Malaysia	Laos	Teachers	Autism	From birth to adolescence, with greater focus on early childhood
Comunidades Inclusivas (Inclusive Communities) module series	Brittany Gregory, Paige Hawkins, Paula Beckman, Don Montagna, Melissa Robbins; Editors: Paula J. Beckman & Don Montagna	2019	English, Spanish	Training modules, each with a training manual and slide presentation	USA	El Salvador	Persons with limited levels of education (high school or less) and for participants who have limited knowledge regarding disability	Learning, behavioural and communication disabilities	18 months—39 years (Beckman and Montagna, 2015a)
Including Children with Autism in Primary Classrooms: A Teacher’s Handbook	Government of India Department of Education of Groups with Special Needs and National Council of Educational Research and Training	2018	English	Handbook	India	India	Regular primary school teachers in inclusive classrooms	Autism	Primary school age
Remedial Learning. Chapter 6 in Moving Away From Labels. Inspired by village school. A Self help Text book on Inclusive Education	CBR Network (South Asia)	2010	English	Handbook	India	India, and more broadly South Asia	Teachers (and parents)	Learning difficulties	Childhood
Communication and Speech Disabilities. Chapter 6 in Moving Away From Labels. Inspired by village school. A Self help Text book on Inclusive Education	CBR Network (South Asia)	2011	English	Handbook	India	India, and more broadly South Asia	Teachers (and parents)	Communication and speech disabilities	Childhood
Inclusion of children with autism—handbook for teachers	The National Trust for the Welfare of Persons with Autism, Cerebral Palsy, Mental Retardation & Multiple Disabilities (Ministry of Social Justice & Empowerment, Govt. of India)	Unavailable	English	Handbook	India	India	Teachers, school management and staff	Autism	Childhood
Including children with special needs—primary stage	Government of India Department of Education of Groups with Special Needs and National Council of Educational Research and Training	2014	English	Handbook	India	India	Regular primary school teachers in inclusive classrooms	ID, Autism	Primary school age
Including children with special needs: upper primary stage	Government of India Department of Education of Groups with Special Needs and National Council of Educational Research and Training	2015	English	Handbook	India	India	Regular upper primary school teachers in inclusive classrooms	ID, Autism	Upper Primary School age
‘Obuntu bulamu’. Peer to peer support for inclusion of children with disabilities—a guide for teachers, training manual for facilitators + training materials	Femke Bannink Mbazzi, Elizabeth Kawesa, Ruth Nalugya, Harriet Nambejja, Claire Nimusiima, Patrick Ojok, Pamela Nizeyimana, Kitaka Mubarak, Aggrey Waguti, Janet Seeley, Geert van Hove, Bongole Wamala	2021	English	Training programme	UK, Uganda	Uganda	Teachers	Autism, ID, communication disabilities, general DD	Childhood
Alertas! De Desarrollo y Estimulación (Warnings! Development and simulation)	Natalia Barrios & Valeria Soto	Unavailable	Spanish	Handbook	Argentina	Argentina	Teachers and technical teams	Autism	16–36 months
Education of Children and Young People with Autism	Rita Jordan	1997	English	Handbook (guide)	United Kingdom	Globally	Teachers, parents, professional groups and community workers	Autism	Childhood (and adolescence and adulthood)
Teaching children with disabilities in inclusive settings	United Nations Educational, Scientific and Cultural Organization (UNESCO) Office Bangkok and Regional Bureau for Education in Asia and the Pacific	2009	English, Arabic, Persian, Bahasa Indonesia	Handbook	Thailand	Asia and the Pacific	Teachers, education planners (and parents)	Learning difficulties, ID, autism, epilepsy, social, emotional and behavioral difficulties	Childhood
Assessing needs (Unit 2 in Understanding and Responding to Children's Needs in Inclusive Classrooms: A Guide for Teachers)	United Nations Educational, Scientific and Cultural Organization (UNESCO)	2001	English, translated in over 20 languages (unspecified)	Unit in a Handbook/ Guide	International	online	Teachers	ID, cerebral palsy	Childhood, school age
Training module on autism spectrum disorders	Indian Government through the Sarva Shiksha Abhiyan (Education for All Movement) programme	Unavailable	English	Training module (handbook)	India	India	Teachers	Autism	Childhood
Educating and caring for children with profound intellectual disability: a manual for carers and teachers	Teacher Empowerment for Disability Inclusion (TEDI) team	2019	English	Handbook (manual)	South Africa	South Africa	Those working and interacting with children with profound intellectual disability, including carers, caregivers, community workers, facilitators, classroom assistants and programme implementers	ID, mentions of other DD	Childhood
Classroom and playground support for children with an autism spectrum disorder	Prithvi Perepa	2014	English	Handbook	UK and South Africa	South Africa	Teachers and school staff with little or no experience of working with children with autism	Autism	Childhood
Environment and surroundings—how to make them autism-friendly	Anh Nguyen	2010	English	Handbook	UK and South Africa	South Africa	Professionals, (parents, and carers)	Autism	Childhood (and adulthood)
Autism preparation kit for teachers	Bec Oakley	2013	English	Handbook (resource kit)	South Africa	South Africa (through Autism South Africa)	Teachers	Autism	Childhood/School-age
Cartilha Autismo & Educação (Autism and Education booklet)	Autismo e Realidade	2013	Portuguese	Handbook	Brazil	Brazil	Teachers	Autism	Primary school

The resources were developed for a range of audiences. Resources listed in Table 1 were primarily directed at the general public or a range of audiences, comprising teachers, health staff and community or religious leaders. Resources in Table 2 mostly targeted non-specialist health workers, including community health workers, general health workers and community childcare workers; some targeted community rehabilitation workers. The education resources (Table 3) targeted a wider range of audiences, including mainstream schoolteachers, parents, school management and community workers.

Three resources were awareness-raising comics or children’s books, 5 were flyers/posters, 2 were fact sheets, 3 were blogs, 6 were videos or video series, 13 were website pages or social media channels featuring several relevant videos (8 resources), flyers/posters (1 resource) or blogs and fact sheets (4 resources). Thirty-two resources were handbooks and self-education guides, one of which could also be used to facilitate a training programme. Of 14 other training programmes or sessions, 1 was an online programme with fully open access, 4 were modules on an interactive app and 9 were to be delivered in person synchronously: for the latter category, the resources retrieved were training manuals, 3 of which had additional materials (e.g., slides).

The comics, children’s books and 10 handbooks focused on reducing stigma in the community or providing health and education workers and community members with general strategies for inclusion. All other handbooks and guides were aimed at teaching strategies to workers in health and education settings and were by far the most common format in this category (accounting for almost the entirety of Tables 2 and 3). Most webpages, videos, and flyers/posters raised awareness on signs, features, and support strategies in multiple stakeholder groups, often including the general public (Table 1).

Around 70% (55 out of 78) of resources had a clear focus on one specific DD. Of these, 41 focused on autism, 10 on intellectual disabilities, 3 on language and communication difficulties, and 1 on epilepsy. Sixteen resources addressed multiple DD, sometimes in combination with other childhood psychosocial disabilities, with specific learning disabilities, or with motor disorders related to conditions, such as cerebral palsy, that are also associated with intellectual disability. Seven resources addressed disabilities more broadly, with relevant sections on autism, intellectual disabilities, and sometimes other DD, such as behavioural and communication disabilities. Most resources taking the latter approach (5) were handbooks or guides for education settings, aimed at including children with disabilities in education. Most resources targeted a wide age range spanning childhood; only a minority of resources focused specifically on early childhood or infancy.

Nineteen resources were developed for and/or used in Africa (mostly Ethiopia and South Africa), 23 in Asia (mostly India and Bangladesh), 24 in Latin America (mostly Argentina and Brazil): all of these except 5 were developed within the context where they were intended to be used, most often by or in partnership with local organisations and experts, and efforts to contextualise to the setting and culture were usually apparent. Twelve resources were developed for cross-continental or global use, although with particular attention to LMICs, as required by our criteria: these were often published by the World Health Organization, UNESCO and other global international agencies, at times with contributions from stakeholders in LMICs. Clear information was usually available on the research evidence supporting the use of resources developed by international agencies and universities and how such resources were evaluated. We often retrieved little or no information on the evidence base for the large number of flyers, blogs and videos and some handbooks/guides developed by smaller organisations. For only a minority of resources was it made explicit that they were developed by or with the involvement of people with DD themselves, and for a few more that they were developed by or with caregivers.

## Discussion

To the best of our knowledge this is the first systematic review of education/training resources on DD for non-specialists in LMICs. Searching the grey literature with targeted methods was especially important due to the nature of the review, that aimed to map resources rather than studies. The strategies used allowed us to identify resources used by several organisations operating in LMICs, including those for which any development and evaluation processes happened informally or were not documented in journals indexed in commonly searched academic databases.

Our mapping analysis revealed that a wealth of materials is available for both global and local use, including comics, children’s books, flyers, posters, fact sheets, blogs, videos, websites pages, social media channels, handbooks and self-education guides, training programmes or sessions. However, a few gaps were identified. First, a large proportion of resources focused on autism, or on specific communication and behavioural challenges, while few resources seemed appropriate to provide non-specialists with a genuine understanding of the needs of children with profound intellectual disabilities. Secondly, while many of the materials identified could be used as part of a training programme, among education resources only few manuals could provide guidance for delivering a full interactive experiential programme, using principles of adult learning [14]. More of such training programmes were available for cadres of health workers. Finally, we did not identify any resources targeted at eastern Europe LMICs, included resources seemed mostly focused on specific countries in Africa (Ethiopia, South Africa), Latin America (Argentina, Brazil) and Asia (India, Bangladesh). While this may be partly due to artefacts of the search (for example because materials from some countries may be more easily discoverable by English language search engines), it should be noted that we actively sought out websites and sources of information from other countries. Studies and materials from other regions, including Eastern Europe, were identified through the search, but rarely or never met criteria for inclusion. Our review thus suggests that training and development for the field of DD is much less prominent in some LMICs than others. This finding mirrors the burgeoning research field on DD in LMIC, where research is steadily growing, but as yet is still concentrated in specific LMICs (Franz et al. 2017) [39]

For a few resources, peer-reviewed articles reporting evaluation studies were available. For example, resources developed for community health workers in Ethiopia were found to reduce negative beliefs and stigmatising attitudes towards autistic children [34]. Similarly, in the Lao People’s Democratic Republic, *The Story of Khamdy* [15], an online learning module on autism, was shown to effectively reduce stigma among schoolteachers who completed the course and increased their knowledge of autism and inclusion in mainstream classes (Low et al. 2021). In Ethiopia, *We’ll Make It*, a comic book on epilepsy increased awareness of community members who read it (specifically, high school students) and its broader distribution was recommended [33]. In the state of Andhra Pradesh in India, *Intellectual Disabilities: A Manual for CBR Workers* [19] was used as part of a broader task-sharing cascade-training intervention which extended coverage of services for children with DD and their families in a sustainable way [20]. These examples show the availability of a few resources evaluated through research studies, in various formats, for various audiences and purposes and on different DDs, that can be used in multiple awareness raising pathways. This is in line with academic literature that recommends awareness-raising campaigns delivered to multiple groups, including family and community-members as well as health and education workers, and both to smal-scale targeted groups and at national and international levels [6, 29].

An identified gap was a scarcity of resources developed with the involvement of people with DD and their families. The call for direct involvement of autistic people and people with intellectual disability in advocacy and research has only relatively recently become more prominent (e.g., [8, 22, 26]), our review suggests this call has not yet resulted in a range of training materials co-developed by the DD community for use in LMIC settings. This is a clear priority for future advocacy and training development efforts. A positive aspect of the resources reviewed is that most of them were developed in the context of their intended use, with the involvement of local clinicians, caregivers, researchers, and educators [11]. Moreover, thanks to the methodology adopted, the resources included in our review do not overlook efforts by grassroots organisations, rarely found in peer-reviewed academic literature. While a downside of this is that several resources have a less clear and formally recorded evaluation, highlighting contributions made to DD knowledge and experience by grassroots organisations is an important strength of our review.

Our review screening process uncovered that several training interventions, both among those developed by NGOs and documented in media articles and those reported in peer-reviewed research reports, were not manualised, or the manual could not be made available for wider use. Such practices are preventing potentially helpful and scalable programmes from being disseminated or even re-used in the same setting where they were developed, after researchers or organisation agents’ departure. We believe this issue should be addressed, in order to promote the scale up of existing programmes and avoid duplicating efforts through time and across countries. While training programmes and interventions should always stem from a local need recognised by stakeholders and be developed within or adapted to the context, several training areas on DD (e.g., the main signs and diagnostic features; [38]) can be largely transferable between LMICs, as adaptations to socio-economic challenges in LMICs compared to high-income countries (e.g., poverty experienced by service users) may be as relevant as cultural adaptations to specific communities [31]. For this reason, we have compiled Tables 1, 2 and 3 and presented all identified resources that can be accessed freely, to support their use in other settings that may need them. We have also provided more detailed information in the additional file, on their content, country of original development and copyright, to encourage researchers and organisations to adopt them, and when possible, adapt them, wherever needed.

## Limitations

The process of our review was designed to systematically identify a large number of resources from both the academic and grey literature, through the use of a variety of methods and comprehensive and systematic search terms. However, a few methodological and practical choices may have reduced our ability to identify all resources.

Some non-English resources were likely missed, as only English search terms were included. However, multiple non-English and non-Western websites and sources were searched and it was noted that at least some of the English search terms were functional to identify resources in those websites: this was especially true for the term “autism”. This may partly explain why the results from our search were slightly skewed towards resources focused on autism compared to other DD. An additional explanation may be the high prevalence of NGOs specifically dedicated to autism identified for inclusion in our search. However, our findings may also reflect a true predominance of resources on autism, in line with previous research that has identified a disproportionally growing interest in research and research funding for autism compared to other DD [4].

Finally, we did not impose date limits on our review and readers should note that there are some outdated resources in our list. Our decisions were guided by the aim to outline all resources available, without quality judgements, to allow other researchers and advocates to select resources most appropriate for their work and context. We provide substantial information on each resource in Tables 1, 2, and 3, to support readers in their resource selection.

## Conclusions

Our systematic review identified a wealth of education and training resources on DD for non-specialists in LMICs, in various formats and with different uses, including raising awareness and training non-specialist professionals in health and education settings. The authors recommend that several resources among the ones described in our results could be employed in different settings to address needs common to other countries or settings compared to where they were originally developed. New interventions and programmes should be made freely accessible and adaptable whenever possible, to increase their impact and reduce the need for new intervention development efforts. Finally, more resource development efforts focused on intellectual disabilities are needed.

### Supplementary Information

**Additional file 1: **Full search strategy [18, 8].

**Additional file 2:**Detailed information on the resources reviewed.

## Data Availability

Not applicable.

## Abbreviations

DD: Developmental disabilities
ID: Intellectual disability (Table 1, 2, 3)
LMIC: Low- and middle-income country

## Footnotes

1 In this manuscript we use a combination of identity-first (for autism) and person-first (for other disabilities) language, to accommodate the spectrum of views of language preferences expressed by self-advocates (Kenny et al., 2016; Bury al., 2020; Buijsman et al., 2022). We have endeavoured to use non-pathologising language throughout (Dwyer et al., 2022) but have used medical terms when referring to review search terms or specific content within the reviewed resources (such as “symptoms” in the additional file) or when directly referring to medical diagnostic criteria.
