# Supplementary material for: Mapping awareness-raising and capacity-building materials on developmental disabilities for non-specialists: a review of the academic and grey literature
Source: Int J Ment Health Syst. 2024 Feb 24;18:10. doi: 10.1186/s13033-024-00627-9 (PMC10893740; doi:10.1186/s13033-024-00627-9)
Supplement: Supplementary file 1 — Additional file 1: Full search strategy [18, 8]. [file 13033_2024_627_MOESM1_ESM.docx]

| **CONTACTING EXPERTS** | | | |
| --- | --- | --- | --- |
| **Experts** | 183 experts overall | |  |
| **Strategy** | - Collate a list of experts from team knowledge and colleagues’ recommendations - Collate contact details and send email request. - Follow up after 2-4 weeks with   - those who have not replied  - those who have replied that they will get back to us   - Follow the same procedure for any subsequently added experts.   Draft email:  “Dear……  We are/I am [sender’s academic role] working on the [name of project] project under the supervision of [last author’s name]. We are currently conducting a systematic review of the grey literature, reviewing existing education and training materials to raise awareness about developmental disorders in the community in low- and middle- income countries.  By building a comprehensive picture of existing training materials that are openly accessible, we hope to inform the development of a training package for communities in Ethiopia and Kenya. As part of our systematic review we are consulting experts like you. Would you mind to let us know if you are aware of any previously developed materials suitable for low-resource contexts relating to raising awareness about child developmental delay, symptoms of developmental disorders, causal explanations, prognosis and/or possible treatments? Examples of the types of resources we are looking for include the training video ‘[recognising autism](https://www.youtube.com/watch?v=lZ1QGW9t8g8)’ developed by Sangath India and [training materials developed for community health workers](https://www.open.edu/openlearncreate/course/view.php?id=2714) in Ethiopia.  We would greatly appreciate any suggestions you may have. Thank you so much for your time.” | |  |
| **ACADEMIC DATABASES** | | | |
| **Databases** | | Ovid MEDLINE, PsycInfo, Global Health, Embase on Ovid (<https://ovidsp.dc1.ovid.com/ovid-b/ovidweb.cgi>) and ERIC on Ebsco (<http://web.a.ebscohost.com/ehost/search/advanced?vid=1&sid=f2a621d8-ad28-4bef-85ff-0fc16fd70d25%40sdc-v-sessmgr02>) | |
| **Links** | | Ovid: <https://ovidsp.dc1.ovid.com/ovid-b/ovidweb.cgi>  ERIC on Ebsco:(<http://web.a.ebscohost.com/ehost/search/advanced?vid=1&sid=f2a621d8-ad28-4bef-85ff-0fc16fd70d25%40sdc-v-sessmgr02> | |
| **Strategy for Ovid** | | - Search terms for all relevant categories combined with AND in Ovid (no use of MESH terms) for the 4 databases simultaneously - Use asterisks for more comprehensive search; no use of “adj” and similar commands for consistency with ERIC - Deduplicate in Ovid | |
| **Strategy for ERIC (Ebsco)** | | - Search terms for all relevant categories combined with AND - Select Boolean/Phrase; no Expanders - Field: run one search for each category where the same category terms are searched with “title” in one box OR “abstract” in other box; then combine searches with AND (so that it’s a title and abstract search like Ovid) - Use asterisks, can’t use adj | |
| **Search Terms** | | Developmental disorder* OR developmental delay* OR developmental disabilit* OR neurodevelopmental disorder* OR neurodevelopmental delay* OR neurodevelopmental disabilit* OR cognitive disabilit* OR cognitive delay* OR intellectual disabilit* OR mental* retard* OR autis* OR Asperger* OR ASD OR learning difficult* OR learning disabilit* OR special needs OR special education needs  **AND**  Education or Training or learning or psychoeducation or teaching* or information or instruction* or guideline* or material* or manual* or professional development  **AND**  Community or non-specialist* or nonspecialist* or layperson* or lay person* or laypeople or lay people or teacher* or teaching assistant* or educator* or school staff or health* worker* or health* extension worker* or health* support worker*  **AND**  Afghanistan or Albania or Algeria or American Samoa or Angola or Argentina or Armenia or Azerbaijan or Bangladesh or Belarus or Byelarus or Byelorussia or Belorussia or Belize or Benin or Bhutan or Bolivia or Bosnia or Herzegovina or Hercegovina or Bosnia-Herzegovina or Bosnia-Hercegovina or Botswana or Brazil or Brasil or Bulgaria or Burkina Faso or Upper Volta or Burundi or Urundi or Cambodia or Republic of Kampuchea or Cameroon or Cameroons or Cape Verde or Cabo Verde or Central African Republic or Chad or China or Colombia or Comoros or Comoro Islands or Comores or Congo or DRC or Zaire or Costa Rica or Cote d'Ivoire or Ivory Coast or Cuba or Djibouti or Obock or Somaliland or Dominica or Dominican Republic or Ecuador or Egypt or United Arab Republic or El Salvador or Eritrea or Ethiopia or Eswatini or Fiji or Gabon or Gabonese Republic or Gambia or Georgia or Ghana or Gold Coast or Grenada or Guatemala or Guinea or Guinea-Bissau or Guiana or Guyana or Haiti or Honduras or India or Indonesia or Iran or Iraq or Jamaica or Jordan or Kazakhstan or Kenya or Kiribati or Korea or DPRK or Kosovo or Kyrgyzstan or Kirghizstan or Kirgizstan or Kirghizia or Kirgizia or Kyrgyz or Kirghiz or Lao or Laos or Lebanon or Lesotho or Basutoland or Liberia or Libya or Macedonia or Madagascar or Malagasy Republic or Malawi or Nyasaland or Malaysia or Malaya or Malay or Maldives or Mali or Marshall Islands or Mauritania or Mayotte or Mexico or Micronesia or Moldova or Moldovia or Mongolia or Montenegro or Morocco or Mozambique or Myanmar or Burma or Namibia or Nepal or Nicaragua or Niger or Nigeria or Pakistan or Palestine or Paraguay or Peru or Philippines or Russia or Russian Federation or USSR or Soviet Union or Union of Soviet Socialist Republics or Rwanda or Ruanda-Urundi or Samoa or Solomon Islands or Sao Tome or Principe or Senegal or Serbia or Montenegro or Yugoslavia or Sierra Leone or Somalia or Sri Lanka or Ceylon or Saint Christopher Island or Saint Lucia or St Lucia or Saint Vincent or St Vincent or Grenadines or Sudan or Suriname or Surinam or Swaziland or Syria or Syrian Arab Republic or Tajikistan or Tadzhikistan or Tadjikistan or Tanzania or Thailand or Timor-Leste or East Timor or Togo or Togolese Republic or Tonga or Tunisia or Turkey or Turkmenistan or Turkmenia or Tuvalu or Uganda or Ukraine or Uzbekistan or Vanuatu or New Hebrides or Venezuela or Vietnam or Viet Nam or West Bank or Gaza or Yemen or Zambia or Zimbabwe or Rhodesia or Africa or Asia or Caribbean or West Indies or Latin America or Central America or South America or Middle East or Eastern Europe or East Europe or third world or LIC or LICs or MIC or MICs or LMIC or LMICs or LAMIC or LAMICs or developing countr* or developing nation* or developing world or developing econom* or developing population* or low-income countr* or low-income nation* or low-income world or low-income econom* or low-income population* or low income countr* or low income nation* or low income world or low income econom* or low income population* or middle-income countr* or middle-income nation* or middle-income world or middle-income econom* or middle-income population* or middle income countr* or middle income nation* or middle income world or middle income econom* or middle income population* or less-developed countr* or less-developed nation* or less-developed world or less-developed econom* or less-developed population* or less developed countr* or less developed nation* or less developed world or less developed econom* or less developed population* or least developed countr* or least developed nation* or least developed world or least developed econom* or least developed population* or under developed countr* or under developed nation* or under developed world or under developed econom* or under developed population* or underdeveloped countr* or underdeveloped nation* or underdeveloped world or underdeveloped econom* or underdeveloped population* or poor countr* or poor nation* or poor world or poor econom* or poor population* or poorer countr* or poorer nation* or poorer world or poorer econom* or poorer population* or transitional countr* or transitional nation* or transitional world or transitional econom* or transitional population* or LAMI countr* or LAMI nation* or LAMI world or LAMI econom* or LAMI population* | |
| **GREY LITERATURE DATABASES** | | | |
| **Databases** | | APA PsycExtra, Eldis, UNESCO library, IRIS (WHO) | |
| **Links** | | APA PsycExtra: <http://0-web.b.ebscohost.com.catalogue.libraries.london.ac.uk/ehost/search/advanced?vid=0&sid=4dc32b07-3433-495d-8169-240a5e446926%40pdc-v-sessmgr06>  Eldis: <https://www.eldis.org/>  UNESCO Library: <https://unesdoc.unesco.org/library>  IRIS: https://apps.who.int/iris/ | |
| **(General) Terms List** | | Developmental disorder OR developmental disorders OR developmental delay OR developmental delays OR developmental disability OR developmental disabilities OR neurodevelopmental disorder OR neurodevelopmental disorders OR neurodevelopmental delay OR neurodevelopmental delays OR neurodevelopmental disability OR neurodevelopmental disabilities OR cognitive disability OR cognitive disabilities OR cognitive delay OR cognitive delays OR intellectual disability OR intellectual disabilities OR mental retardation OR special needs OR special education needs OR learning difficulty OR learning difficulties OR learning disability OR learning disabilities OR autism OR autistic OR ASD OR Asperger | |
| **Strategy for APA PsycExtra**  **(Ebsco)** | | - DD terms AND Country terms   - Do not use quotation marks.   - 2 categories; all terms for one category entered into one search bar, separated by “OR” in between each term. The two boxes are combined with AND   - “Select a field (optional)” is not selected. - Export to Excel - Remove duplicates, only selecting the numbers column [https://support.microsoft.com/en-us/office/find-and-remove-duplicates-00e35bea-b46a-4d5d-b28e-66a552dc138d (the second part of the page is to remove them) In the Remove Duplicates box, only select the column with the numbers]   Search terms:  Box 1: Developmental disorder OR developmental disorders OR developmental delay OR developmental delays OR developmental disability OR developmental disabilities OR neurodevelopmental disorder OR neurodevelopmental disorders OR neurodevelopmental delay OR neurodevelopmental delays OR neurodevelopmental disability OR neurodevelopmental disabilities OR cognitive disability OR cognitive disabilities OR cognitive delay OR cognitive delays OR intellectual disability OR intellectual disabilities OR mental retardation OR special needs OR special education needs OR learning difficulty OR learning difficulties OR learning disability OR learning disabilities OR autism OR autistic OR ASD OR Asperger  AND  Box 2: Afghanistan or Albania or Algeria or American Samoa or Angola or Argentina or Armenia or Azerbaijan or Bangladesh or Belarus or Byelarus or Byelorussia or Belorussia or Belize or Benin or Bhutan or Bolivia or Bosnia or Herzegovina or Hercegovina or Bosnia-Herzegovina or Bosnia-Hercegovina or Botswana or Brazil or Brasil or Bulgaria or Burkina Faso or Upper Volta or Burundi or Urundi or Cambodia or Republic of Kampuchea or Cameroon or Cameroons or Cape Verde or Cabo Verde or Central African Republic or Chad or China or Colombia or Comoros or Comoro Islands or Comores or Congo or DRC or Zaire or Costa Rica or Cote d'Ivoire or Ivory Coast or Cuba or Djibouti or Obock or Somaliland or Dominica or Dominican Republic or Ecuador or Egypt or United Arab Republic or El Salvador or Eritrea or Ethiopia or Eswatini or Fiji or Gabon or Gabonese Republic or Gambia or Georgia or Ghana or Gold Coast or Grenada or Guatemala or Guinea or Guinea-Bissau or Guiana or Guyana or Haiti or Honduras or India or Indonesia or Iran or Iraq or Jamaica or Jordan or Kazakhstan or Kenya or Kiribati or Korea or DPRK or Kosovo or Kyrgyzstan or Kirghizstan or Kirgizstan or Kirghizia or Kirgizia or Kyrgyz or Kirghiz or Lao or Laos or Lebanon or Lesotho or Basutoland or Liberia or Libya or Macedonia or Madagascar or Malagasy Republic or Malawi or Nyasaland or Malaysia or Malaya or Malay or Maldives or Mali or Marshall Islands or Mauritania or Mayotte or Mexico or Micronesia or Moldova or Moldovia or Mongolia or Montenegro or Morocco or Mozambique or Myanmar or Burma or Namibia or Nepal or Nicaragua or Niger or Nigeria or Pakistan or Palestine or Paraguay or Peru or Philippines or Russia or Russian Federation or USSR or Soviet Union or Union of Soviet Socialist Republics or Rwanda or Ruanda-Urundi or Samoa or Solomon Islands or Sao Tome or Principe or Senegal or Serbia or Montenegro or Yugoslavia or Sierra Leone or Somalia or Sri Lanka or Ceylon or Saint Christopher Island or Saint Lucia or St Lucia or Saint Vincent or St Vincent or Grenadines or Sudan or Suriname or Surinam or Swaziland or Syria or Syrian Arab Republic or Tajikistan or Tadzhikistan or Tadjikistan or Tanzania or Thailand or Timor-Leste or East Timor or Togo or Togolese Republic or Tonga or Tunisia or Turkey or Turkmenistan or Turkmenia or Tuvalu or Uganda or Ukraine or Uzbekistan or Vanuatu or New Hebrides or Venezuela or Vietnam or Viet Nam or West Bank or Gaza or Yemen or Zambia or Zimbabwe or Rhodesia or Africa or Asia or Caribbean or West Indies or Latin America or Central America or South America or Middle East or Eastern Europe or East Europe or third world or LIC or LICs or MIC or MICs or LMIC or LMICs or LAMIC or LAMICs or developing countries or developing nations or developing world or developing economies or developing populations or low-income countries or low-income nations or low-income world or low-income economies or low-income populations or low income countries or low income nations or low income world or low income economies or low income populations or middle-income countries or middle-income nations or middle-income world or middle-income economies or middle-income populations or middle income countries or middle income nations or middle income world or middle income economies or middle income populations or less-developed countries or less-developed nations or less-developed world or less-developed economies or less-developed populations or less developed countries or less developed nations or less developed world or less developed economies or less developed populations or least developed countries or least developed nations or least developed world or least developed economies or least developed populations or under developed countries or under developed nations or under developed world or under developed economies or under developed populations or underdeveloped countries or underdeveloped nations or underdeveloped world or underdeveloped economies or underdeveloped populations or poor countries or poor nations or poor world or poor economies or poor populations or poorer countries or poorer nations or poorer world or poorer economies or poorer populations or transitional countries or transitional nations or transitional world or transitional economies or transitional populations or LAMI countries or LAMI nations or LAMI world or LAMI economies or LAMI populations | |
| **Strategy for Eldis** | | - Conduct searches the DD terms, with only 1 term in the search box at a time (because when searching more than one term, Eldis will only search the last term written). This means each search term will be searched independently (i.e., you will not search 5 terms in one search box).   - Search without quotation marks.   - Only singular or plural   - Terms with two words, will use dash, for example cognitive-disabilities   [because:  -If you use AND it will search up both terms separately (i.e., results for “cognitive” and results for “disabilities”, but not as one phrase/term).  -If you use dash (cognitive-disabilities) it will search the phrase.]   - Export the search results manually for each independent search (copy and paste in google spreadsheet).   Search terms:  Developmental-disorder  Developmental-delay  Cognitive-disability  Intellectual-disability  Developmental-disability  Neurodevelopmental-disorder  Autism  Autistic  Special-needs  Learning-difficulty  Mental-retardation  Asperger  Neurodevelopmental-delay  Neurodevelopmental-disability  Cognitive-delay  Special-education-needs  Learning-disability  ASD | |
| **Strategy for UNESCO Library** | | - Conduct search with new DD terms, selecting “Boolean expression” (to allow for the use of OR) and selecting “title”   - Enter all terms into one search bar and separate them by OR.   - Use quotations for each term because this is necessary for the database to search the two words together. - Export in Excel   Search terms:  “Developmental disorder” OR “developmental disorders” OR “developmental delay” OR “developmental delays” OR “developmental disability” OR “developmental disabilities” OR “neurodevelopmental disorder” OR “neurodevelopmental disorders” OR “neurodevelopmental delay” OR “neurodevelopmental delays” OR “neurodevelopmental disability” OR “neurodevelopmental disabilities” OR “cognitive disability” OR “cognitive disabilities” OR “cognitive delay” OR “cognitive delays” OR “intellectual disability” OR “intellectual disabilities” OR “mental retardation” OR “special needs” OR “special education needs” OR “learning difficulty” OR “learning difficulties” OR “learning disability” OR “learning disabilities” OR autism OR autistic OR ASD OR Asperger | |
| **Strategy for IRIS (WHO)** | | - Enter DD terms in the search box as follows:   - Enter all terms into one search bar and separate them by OR.   - Use quotations for each term because this is necessary for the database to search the two words together. - Add filter one by one, one for each DD term: for each of them, select “Title” and “Contain”, enter search term (no quotation marks, slashes or parentheses), apply, click “Export”, select Excel, then remove filter and do it all again with the next term - Merge Excel file   Search terms:  “Developmental disorder” OR “developmental disorders” OR “developmental delay” OR “developmental delays” OR “developmental disability” OR “developmental disabilities” OR “neurodevelopmental disorder” OR “neurodevelopmental disorders” OR “neurodevelopmental delay” OR “neurodevelopmental delays” OR “neurodevelopmental disability” OR “neurodevelopmental disabilities” OR “cognitive disability” OR “cognitive disabilities” OR “cognitive delay” OR “cognitive delays” OR “intellectual disability” OR “intellectual disabilities” OR “mental retardation” OR “special needs” OR “special education needs” OR “learning difficulty” OR “learning difficulties” OR “learning disability” OR “learning disabilities” OR autism OR autistic OR ASD OR Asperger  Filters applied to Title:  Developmental disorder  Developmental delay  Developmental disability  Neurodevelopmental disorder  Neurodevelopmental delay  Neurodevelopmental disability  Cognitive disability  Cognitive delay  Intellectual disability  Mental retardation  Special needs  Special education needs  Learning difficulty  Learning disability  Autism  Autistic  Asperger  ASD | |
| **CUSTOMISED GOOGLE SEARCHES** | | | |
| **Search Engines** | | 17 Customised Google Search Engines   1. <https://cse.google.com/cse?cx=668c20f1ab6d74972> 2. <https://cse.google.com/cse?cx=558389854d0e59334> 3. <https://cse.google.com/cse?cx=e4308a2c6cd267801> 4. <https://cse.google.com/cse?cx=7b97674c500576b15> 5. <https://cse.google.com/cse?cx=907693c6d244500e6> 6. <https://cse.google.com/cse?cx=2c9666bc1a7f595c7> 7. <https://cse.google.com/cse?cx=c9c26132f721274f3> 8. <https://cse.google.com/cse?cx=f834bd1cce038d85f> 9. <https://cse.google.com/cse?cx=679e0ddb5797bcdd2> 10. <https://cse.google.com/cse?cx=7256401b73940a298> 11. <https://cse.google.com/cse?cx=52c18d755ee2719db> 12. <https://cse.google.com/cse?cx=b470d6c92164656e2> 13. <https://cse.google.com/cse?cx=94ce0f1a630561a14> 14. <https://cse.google.com/cse?cx=7486ee7f790e37177> 15. <https://cse.google.com/cse?cx=4f8b8d4a993e8be94> 16. <https://cse.google.com/cse?cx=943f61ca70acbcc0a> 17. <https://cse.google.com/cse?cx=d11745fc49adc70b1> | |
| **Strategy** | | Strategy Instructions:   - Search DD terms using inverted commas - Extract by copying and pasting in google spreadsheet, then copy to Excel   Search terms:  “Developmental disorder” OR “developmental disorders” OR “developmental delay” OR “developmental delays” OR “developmental disability” OR “developmental disabilities” OR “neurodevelopmental disorder” OR “neurodevelopmental disorders” OR “neurodevelopmental delay” OR “neurodevelopmental delays” OR “neurodevelopmental disability” OR “neurodevelopmental disabilities” OR “special needs” OR “autism” OR “autistic” OR ASD OR Asperger OR “special education needs” OR “learning difficulty” OR “learning difficulties” OR “learning disability” OR “learning disabilities” OR “cognitive disability” OR “cognitive disabilities” OR “cognitive delay” OR “cognitive delays” OR “intellectual disability” OR “intellectual disabilities” OR “mental retardation” | |
